# Addressing planetary health challenges in Africa

**DOI:** 10.1186/s40985-016-0046-z

**Published:** 2016-11-29

**Authors:** Alex Ezeh

**Affiliations:** 1 African Population and Health Research Center, Nairobi, Kenya; 2 School of Public Health, University of the Witwatersrand, Johannesburg, South Africa

**Keywords:** Planetary health, Human health, Africa, Climate change, Population growth, Natural resource management, Urbanization

## Abstract

Drawing on the report of the Rockefeller Foundation-Lancet Commission on planetary health—Safeguarding human health in the Anthropocene epoch, this piece presents a discussion of the implications of the report’s findings and conclusions for Africa. It explores the key planetary health challenges facing Africa and what Africa can do to address them. In addition to highlighting current and future trajectories of key environmental changes in Africa and their implications for health and well-being, this transcript from the 21st Conference of Parties (COP21) side event, “Healthy Lives on a Healthy Planet”, identifies a set of priority action Africa needs to take in order to deal with these challenges. It ends with reflections and key recommendations from participants at the regional launch of the report in Nairobi, Kenya, in October 2015.

## Background

Professor Haines, chair of the Rockefeller Foundation-Lancet Commission on Planetary Health, has done an excellent job providing a high-level summary of the commission’s report on *Safeguarding human health in the Anthropocene epoch* [1]. My aim is to examine what this report really means for Africa. As it is commonly known, Africa contributes the least to global climate change but bears disproportionate burden of the adverse consequences of climate change [2]. Rather than focus on the various ways, Africa remains exposed to the consequences of climate change, I will focus on three key drivers of global environmental change where an African response could make a huge difference and limit the continent’s exposure in the longer term.

## Main Text

One of the key drivers of environmental change globally is population growth. In 1950, Africa accounted for about 9% of the world population; by 2100, it is estimated to account for about 40% of the world population, with a projected total population of 4.4 billion people [3, 4]. Indeed, 83% of the projected increase in global population by 2100 will occur in Africa. When we think of a world with 4.4 billion Africans, it may give us a chill for many different reasons. The real question, though, is what type of 4.4 billion people are we going to have in Africa by 2100? Is it going to be 4.4 billion poorly educated, hungry, and sick people trying to leave the continent for greener pastures elsewhere or will it be 4.4 billion well-educated, healthy, and productively engaged citizens contributing to the development of the region? I think these are the critical questions that we should engage with now. The current and projected rates of population growth in Africa make it difficult for many governments in the region to make the necessary investments in human capital development needed to transform the region.

While the projected population of 4.4 billion Africans by 2100 has some validity, it is not necessarily a predetermined and inevitable destiny for the continent. Recent examples from Ethiopia and Rwanda assure us that significant disruptions in fertility levels and population growth rates can occur within a very short time period under the right policy and programmatic contexts [5, 6]. Most countries in Africa are ready for such significant changes in reproductive norms. One in four women in Africa still has an unmet need for family planning [7]. Responding to and meeting this potential demand can significantly reduce the region’s population growth rate, especially unplanned pregnancies which account for nearly 40% of all pregnancies in Africa [8]. Another opportunity for Africa to change this demographic future is to increase age at first marriage and first childbearing. Over the last 60 years, age at first marriage has not really changed much in most of Africa, especially among rural populations where mean age at first marriage for women is still below the age of 18 [9]. Increasing age at first marriage can improve female education in the short term and reduce population growth in the medium and longer term by increasing the gap between generations. Finally, increasing access to female education will have immediate and longer term effects on slowing population growth. Most of these are cost-effective, easily implementable policy options that can significantly change the course of population growth in Africa. Not addressing Africa’s continuing rapid rate of population growth limits the capacity of governments to make necessary investments in human capital development which, in turn, forces increasingly large numbers of people in Africa to depend primarily on the provisioning services of already fragile and degraded ecosystems. Given the small environmental footprint of many African countries, any efforts to slow population growth rates in Africa must be matched by appropriate, complementary efforts to mitigate the environmental damage brought by countries with the heaviest environmental footprints, even if they are experiencing zero or negative population growth.

The second major aspect of the Planetary Health report that is critically relevant for Africa is the management of Africa’s natural resources and ecosystems. The issue of degradation of the natural environment and ecosystems is a major challenge for Africa. Africa currently suffers from deforestation that is at least twice the rate of the world average [10]. In West Africa, the estimate is that about 90% of original forests have already been deforested. Africa lost the highest percentage of tropical forests of any continent during the last three decades. Similarly, land degradation in the past three decades has been very high due to expansion of agriculture and changing land use [11]. Changing land use, deforestation, desertification, and land degradation are already having and are expected to continue to have impacts on environment and health status in Africa. For example, malaria transmission is now evident in many areas where it was previously absent. Over the years, improvements in agriculture in Africa have largely been driven by expansion of land area being cultivated rather than by increasing yield per acre. Many countries have already run out of space in terms of increasing the land area that could be cultivated. About 95% of agriculture in Africa is still rain-fed, and about 70% of arable land is degraded [12]. In 37 African countries, severe soil-nutrient depletion over the past 30 years has led to substantial soil impoverishment and reduced agricultural output [13]. The constellation of these factors poses real challenges for Africa and raises a number of fundamental questions regarding the prospects of social cohesion and food security in the region. Forest and land conservation policies for greener and healthy Africa are urgently needed [14]. A number of organizations are already actively engaged in addressing these issues including African Wildlife foundation, African Conservation Center, African Rainforest Conservancy, African Biodiversity Network, among others. These efforts need to be sustained and expanded for greater impact. Also needed are integrated strategies to address growing demands for food within environmental limits through food and agricultural policies such as sustainable intensification, efficient use of water and fertilizer, reduction of food wastage and spoilage, sustainable aquaculture and fisheries, support for subsistence farmers, innovative sources of nutrition, promotion of healthy, low environmental impact diets, and promotion of environmentally friendly alternatives to wood fuel.

The third key area of the report that Africa cannot afford to ignore is the role of urbanization. Although Africa is still the least urbanized region of the world, it is the most rapidly urbanizing region. Many cities are projected to continue to grow at rates of more than 7% over the coming decades. The share of Africans living in urban areas soared from 15% in 1960 to 40% in 2010 and is projected to grow to 60% by 2050 [15]. Currently, about 60% of urban populations in Africa live in slums or informal settlements [16]. As smaller towns grow into cities, without proper planning and provision of basic amenities, especially with devolved systems of government creating new centers of attraction, slums will proliferate. Work by the African Population and Health Research Center (APHRC) has shown that morbidity, access to health services, and mortality rates are worse for slum residents than for any other subgroup [17]. Whether this rapid rate of urbanization can lead to economic growth, transformation, and poverty reduction or to increased inequality, growing urban poverty, and the proliferation of slums remains an unanswered question. It is however clear that Africa cannot effectively address its growth and poverty challenges nor deal with the environmental consequences of these without addressing and managing its rate of urbanization. The use of environmental friendly technology along with good urban planning would play a significant role in addressing these impacts. It should be noted that urbanization is not a subplot, but rather the main policy narrative for Africa, now and in the future.

## Conclusions

There are many other issues raised in the report that are relevant for Africa but I will devote this last section to views from a regional dissemination of the report in Nairobi in October 2015. The participants at this event were drawn from academia, civil society, regional and national policy makers, youth groups, and experts in the health and climate change fields. The participants unanimously endorsed the recommendation that action is needed at all levels to address the issues of planetary health in Africa and globally. The magnitude of the challenge and the severity of the consequences demand individual responsibility and action at household, community, local authority, national government, and regional/continental body levels. The group underscored the need for a multilayered action plan to implement a planetary health agenda in Africa. They identified a number of priority areas where action is needed, including research and training priorities and policy and governance priorities. They underscored the role of partnerships and regional cooperation in addressing these challenges. They also noted that global processes and agreements on climate change need to connect better with what people think and do in their households and local communities in order to achieve a healthy balance between our environment (planet) and our population (people).

## Abbreviations

APHRC: African Population and Health Research Center
